# ADH5 inhibits proliferation but promotes EMT in non-small cell lung cancer cell through activating Smad2/Smad3

**DOI:** 10.1515/biol-2025-1148

**Published:** 2025-09-23

**Authors:** Xinyu Tan, Ye Huang, Xiaolei Li, Fei Xu, Xinping Xu

**Affiliations:** Jiangxi Institute of Respiratory Disease, The Department of Respiratory and Critical Care Medicine, Jiangxi Clinical Research Center for Respiratory Diseases, The First Affiliated Hospital, Jiangxi Medical College, Nanchang University, Nanchang, 330006, China; Jiangxi Hospital of China-Japan Friendship Hospital, Nanchang, 330200, China; Jiangxi Provincial Key Laboratory of Respiratory Diseases, Nanchang, 330006, China

**Keywords:** ADH5, NSCLC, Smad2/Smad3, proliferation, EMT

## Abstract

Cell proliferation and epithelial–mesenchymal transition (EMT) are two common tumor phenotypes closely linked to malignant tumor progression. Genes regulating tumor progression often exhibit consistent regulatory trends in these phenotypes; however, certain genes may display inconsistent regulatory patterns in tumor proliferation and EMT. In this investigation, initial transcriptomic and tumor database analyses revealed that alcohol dehydrogenase 5 (ADH5) is downregulated in non-small cell lung cancer (NSCLC) tissues and correlates negatively with NSCLC prognosis. Subsequent experimental manipulation of ADH5 levels in tumor cells demonstrated that ADH5 overexpression decreased proliferation while enhancing migration and invasion capacities in NSCLC cells. Moreover, ADH5 overexpression hindered xenograft tumor growth in nude mice. However, ADH5 knockdown yielded contrasting outcomes by stimulating NSCLC cell proliferation while impeding migration and invasion abilities. Notably, ADH5 overexpression triggered EMT through Smad2/Smad3 activation, leading to the upregulation of SRY-Box Transcription Factor 9. TGFbetaR1/ALK5 inhibitor SB431542 was able to alleviate the effects of ADH5 overexpression on NSCLC cells. This study indicates a critical role of ADH5 in tumors associated with cancer cell growth inhibition but EMT activation. These findings underscore ADH5 as a potential regulator of NSCLC cell plasticity, emphasizing its promise as a therapeutic target for NSCLC management.

## 1 Introduction

Non-small cell lung cancer (NSCLC), the predominant histological subtype of lung cancer, ranks as the second most common and deadly cancer globally [1]. Although significant advances have been made in the treatments of NSCLC, including targeted therapy and immune therapy, the prognosis still falls short due to the ongoing evolution of cancer cells. Despite the positive response to treatment at the beginning, the eventual emergence of drug resistance and immune evasion leads to the malignancy of cancer cells. Unravelling the mechanisms through which cancer cells modulate their phenotypes to gain advantages in motility and proliferation during cancer progression remains a challenge.

Alcohol dehydrogenase 5 (ADH5) is a member of the alcohol dehydrogenase family and responsible for metabolizing intracellular S-nitrosoglutathione, which indirectly regulates protein S-nitrosylation [2]. By reducing protein S-nitrosylation, ADH5 functions as an antagonist of nitric oxide, a molecule is known for its dual roles in biological processes and tumorigenesis [3,4]. Formaldehyde dehydrogenase and ADH5 were discovered to be one enzyme in 1989 [5]. Aldehyde-driven DNA damage leading to AMeD syndrome and Cockayne syndrome, has been found to be the consequence of digenic mutations in ADH5 and aldehyde dehydrogenase 2 or Cockayne syndrome protein B [6,7]. The modulation of S-nitrosylation and formaldehyde by ADH5 has been implicated in various fields including hepatocellular carcinoma, cardiovascular diseases, and senescence [8–17], and mainly functions as a protector of the cell homeostasis. However, the integral role of ADH5 in the progression of NCSLC still remains to be elucidated.

Here, this study revealed that ADH5 is downregulated in NSCLC tissues and cell lines. ADH5 overexpression resulted in reduced proliferation but increased migration and invasion of NSCLC cells; whereas, ADH5 knockdown led to enhanced proliferation and reduced migration and invasion. Further investigations demonstrated that overexpression of ADH5 activated Smad2/Smad3 signaling and induced epithelial–mesenchymal transition (EMT) in NSCLC cells. We then applied TGFbetaR1/ALK5 inhibitor SB431542 to rescue the consequence of ADH5 overexpression confirming the regulation of Smad2/3 by ADH5. Moreover, a positive correlation was observed between ADH5 and SRY-Box Transcription Factor 9 (SOX9), with ADH5 upregulating SOX9 expression in NSCLC cells. These findings suggest that ADH5 may serve as a novel regulator of cancer cell plasticity in NSCLC.

## 2 Materials and methods

### 2.1 Tissue samples and mice

NSCLC tissues and paired adjacent tissues were collected from patients who were histologically diagnosed at First Affiliated Hospital of Nanchang University between 2020 and 2022. All patients signed informed consent for the research. This study has been approved by the Human Ethics Committee of First Affiliated Hospital of Nanchang University (CDYFYYLK(10-008)). Balb/c nude mice were housed in a pathogen-free laboratory animal center at First Affiliated Hospital of Nanchang University. Animal experiments were scrutinized and approved (CDYFY-IACUC-202210QR002).


**Informed consent:** Informed consent has been obtained from all individuals included in this study.
**Ethical approval:** The research related to human use has been complied with all the relevant national regulations, institutional policies and in accordance with the tenets of the Helsinki Declaration, and has been approved by the Human Ethics Committee of First Affiliated Hospital of Nanchang University (CDYFYYLK(10-008)).
**Ethical approval:** The research related to animal use has been complied with all the relevant national regulations and institutional policies for the care and use of animals, and has been approved by the Ethical Committee of The First Affiliated Hospital of Nanchang University (CDYFY-IACUC-202210QR002).

### 2.2 Tumor formation in nude mice

PC9 cells overexpressing ADH5 and PC9 cells containing the relative empty vector were prepared in 100 µL with 4 × 10^6^ cells and injected subcutaneously into male BALB/c nude mice. Tumor volumes were measured and calculated every 3 days according to the formula *V*  =  0.5  ×  *L*  ×  *W* ^2^. Five weeks later, the nude mice were sacrificed and the tumors were collected for further analysis.

### 2.3 Cell cultures

The NSCLC cell lines and normal human bronchial epithelial cell line were purchased from ATCC. Cells were cultured in RPMI-1640 (Gibco) or Dulbecco’s modified Eagle’s medium (Gibco) supplemented with 10% fetal bovine serum (FBS), 100 U/mL penicillin, and 100 mg/mL streptomycin in incubators at 37°C with 5% carbon dioxide.

### 2.4 Construction of stable cell lines

H358 and PC9 cells were transfected with ADH5 expression plasmids (Origene) using lipofectamine 3000 (Invitrogen) according to the manufacturer’s instructions. Two days after the transfection, neomycin was applied for a week. Limited dilution assay was performed to acquire monoclonal ADH5 overexpression cells. Validation of overexpression via western blot confirmed two H358 monoclonal overexpression cell lines and one PC9 monoclonal overexpression cell line. shRNA plasmids for ADH5 and corresponding negative control (Origene) were transfected into the 293 T cell. After 48 h incubation, virus-containing supernatants were collected for transfection into H358 and PC9 cells. Puromycin was applied for 3 days. Knock-down was confirmed by western blot.

### 2.5 qRT-PCR

TRIzol reagent (TransGen Biotech) was used to extract total RNA from cells and the purity and integrity were assessed by Nanodrop 2000/2000C spectrophotometry (Thermo Fisher Scientific). Total RNA was reversely transcribed to high-quality cDNA using All-in-One First-Strand cDNA Synthesis SuperMix for qPCR (TransGen Biotech). qRT-PCR was performed using SYBR Green Mix (Thermo Fisher Scientific). Results were normalized to the expression of glyceraldehyde-3-phosphate dehydrogenase (GAPDH) (Table 1).

**Table 1:** Primers used in qRT-PCR

Gene	Forward (5′–3′)	Reverse (5′–3′)
GAPDH	ACCACAGTCCAT GCCATCAC	TCCACCACCCTGTTGCTGTA
ADH5	ATGGCGAACGAGGTTATCAAG	CATGTCCCAAGATCACTGGAAAA
c-Myc	TCTCCTTGCAGCTGCTTAG	GTCGTAGTCGAGGTCATAG
P15	CACCGTTGGCCGTAAACTTAAC	TAATGAAGCTGAGCCCAGGTCT

### 2.6 Western blotting

Total proteins were collected with ice-cold RIPA lysis buffer and the concentrations were determined by BCA protein reagent kit. Then the proteins were heated at 100°C with protein loading buffer. Samples were subjected to SDS-PAGE and transferred to PVDF membrane. The antibodies used in the study include: β-actin antibody (1/5,000; Proteintech), ADH5 antibody (1/2,000; Abcam), SMAD2/3 antibody (1/5,000; Cell Signaling Technology), phospho-SMAD2/3 (1/2,000; Cell Signaling Technology), E-cadherin antibody (1/2,000; Cell Signaling Technology), N-cadherin antibody (1/2,000; Cell Signaling Technology), Snail antibody (1/2,000; Cell Signaling Technology), Slug antibody (1/2,000; Cell Signaling Technology), AKT antibody (1/5,000; Proteintech), p-AKT antibody (1/2,000; Proteintech), and SOX9 antibody (1/2,000; Proteintech).

### 2.7 Assays for CCK8 cell proliferation and colony formation

H358 and PC9 cells with knocked down or overexpressed ADH5 were seeded onto 96-well plates (1 × 10^3^ cells per well). CCK8 (Solarbio) was used according to the manufacturer’s instructions. For colony formation, cells were seeded at a density of 1.0 × 10^3^ cells/mL in six-well plates. After 2 weeks’ culture, cells were fixed with 4% paraformaldehyde and stained with crystal violet (Solarbio).

### 2.8 Transwell assays

Transwell membranes (Corning Costar) were used. For migration assays, 4.0 × 10^4^ cells in 200 μL of RPMI-1640 medium containing 0.1% FBS were added to the upper chambers of transwell plates. Medium containing 10% FBS was added to the bottom chamber. After incubation, the migrated cells were fixed with 4% paraformaldehyde and stained with crystal violet. For invasion assay, 5.0 × 10^4^ cells were seeded to the top chambers coated with Matrigel. After 36 h, the cells were fixed with 4% paraformaldehyde and stained with crystal violet.

### 2.9 Bioinformatics of public databases

The expression of ADH5 in LUAD was obtained from the Cancer Genome Atlas (TCGA) data visualization web-tool UALCAN (http://ualcan.path.uab.edu/). ADH5 protein expression in normal lung tissues and lung adenocarcinoma was from The Human Protein Atlas (THPA) database (https://www.proteinatlas.org/). Kaplan–Meier analysis of ADH5 in LUAD was acquired from Kaplan–Meier Plotter (http://kmplot.com/). The correlation between ADH5 and SOX9 was obtained from Gepia database (http://gepia.cancer-pku.cn/).

### 2.10 Statistical analysis

GraphPad Prism 8.0 was used to perform statistical analysis. Numerical data were expressed as mean ± standard deviation. Statistical significance was defined as *P*  <  0.05. Student’s *t*-test and one-way analysis of variance were used to compare the expression levels of two groups and more, respectively.

## 3 Results

### 3.1 ADH5 was downregulated in NSCLC tissues and cell lines

**Figure 1:**
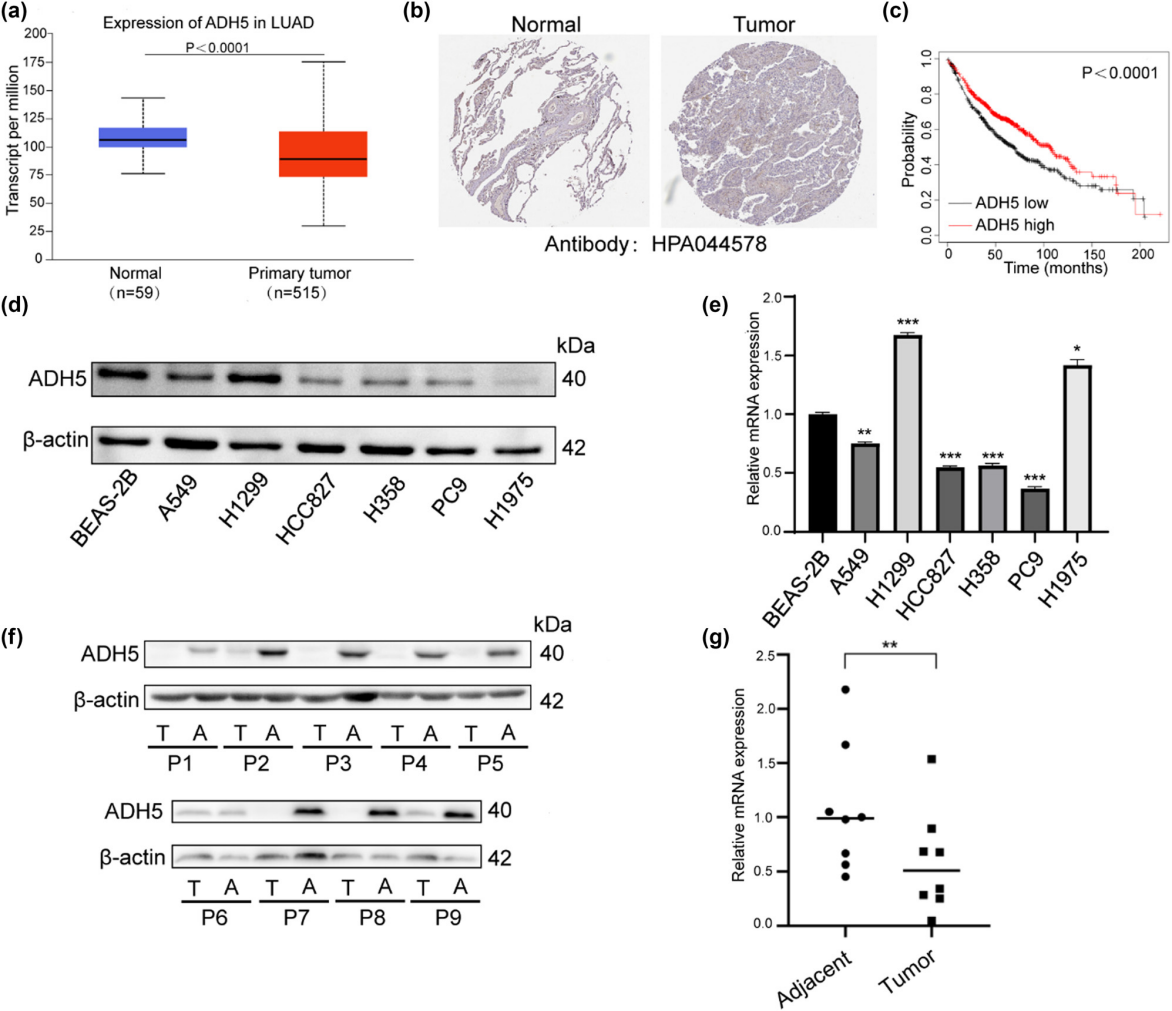
Expression of ADH5 in NSCLC cell lines and tissues. (a) mRNA expression of ADH5 from TCGA database. (b) ADH5 protein expression in normal tissues and lung adenocarcinoma from THPA. (c) Kaplan–Meier analysis indicated that the low expression of ADH5 was significantly correlated with poor overall survival. (d) Western blotting analysis of ADH5 expression in NSCLC cell lines and one normal human bronchial epithelial cell line. (e) Relative mRNA expression of ADH5 in lung cancer cell lines. ADH5 expression was examined using qRT-PCR and was normalized to GAPDH expression. (f) Western blotting analysis of ADH5 expression in NSCLC tissues compared with corresponding adjacent tissues. (g) Relative expression levels of ADH5 in NSCLC tissues compared with corresponding adjacent tissues. Data from cells were generated from at least three biologically independent experiments. *P < 0.05, **P < 0.01, ***P < 0.001.

First, we conducted a bioinformatic analysis to examine the expression of ADH5 in NSCLC. Data from the TCGA database indicated a decrease in ADH5 mRNA levels in lung adenocarcinoma (Figure 1a). The protein levels of ADH5 also decreased in the THPA database (Figure 1b), which has been reported in several types of cancers [8,18–20]. Furthermore, the survival probability of LUAD patients with high ADH5 expression was significantly higher than those with low ADH5 expression level (Figure 1c). Subsequently, to validate both the mRNA and protein expression of ADH5, western blot and qRT-PCR were conducted to confirm ADH5 expression in normal cell line and NSCLC cell lines (Figure 1d and e). When compared to the normal human bronchial epithelial cell line BEAS-2B, both the protein and mRNA levels of ADH5 were significantly downregulated in NSCLC cell lines, except H1299. The loss of p53 protein in H1299 may contribute to ADH5 overexpression because p53 directly regulates ADH5 [21]. We further confirmed the mRNA and protein expression of ADH5 in paired NSCLC tissues, revealing lower levels of both mRNA and protein in the NSCLC tissues than that in adjacent normal tissues (Figure 1f and g), given that ADH5 has been reported to be post-transcriptionally regulated [21]. Significant downregulation of mRNA level and protein level of ADH5 were found in both lung cancer cell lines and tissues. These findings imply that ADH5 is downregulated in NSCLC and could potentially contribute to the pathogenesis of the disease.

### 3.2 ADH5 overexpression impaired proliferation but enhanced migration and invasion of NSCLC cells

**Figure 2:**
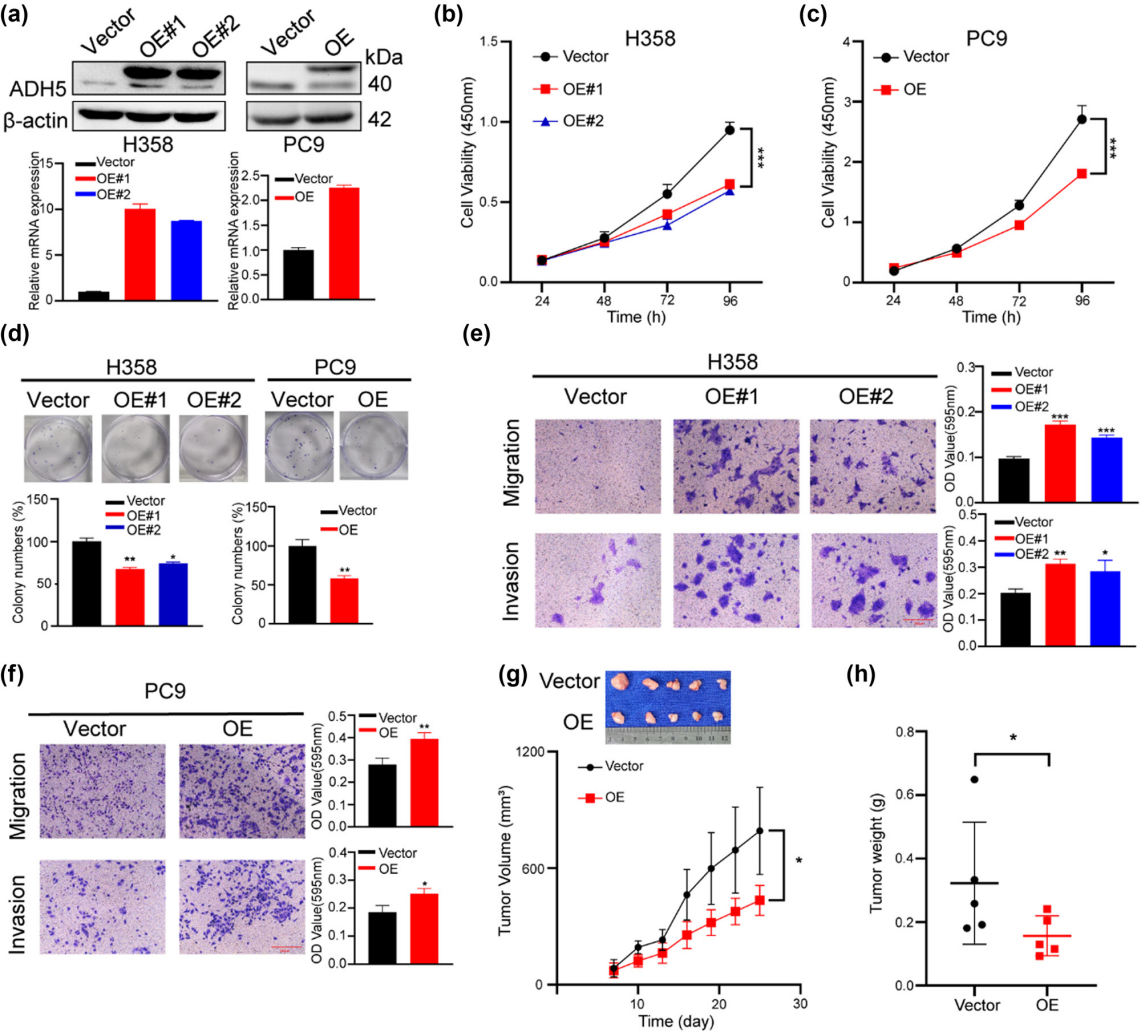
ADH5 overexpression impaired proliferation but enhanced metastasis of NSCLC cells. (a) Western blot and qRT-PCR showed that ADH5 was efficiently overexpressed in H358 and PC9 cells. (b) and (c) Cell-viability assay was performed to measure the growth of ADH5-transfected H358 and PC9 cells. (d) Colony formation was conducted in H358 and PC9 cells transfected with or without ADH5. (e) and (f) Transwell assays were performed to evaluate migration and invasion of ADH5 overexpressed H358 cells and PC9 cells. For migration H358 was incubated for 24 h, PC9 was incubated for 24 h. For invasion H358 was incubated for 48 h, PC9 was incubated for 36 h. (g) and (h) PC9 ADH5-overexpressed cells were subcutaneously injected into BALB/c nude mice. Tumor volumes were evaluated every 3 days. Tumor weights were evaluated 4 weeks later. Data from cells were generated from at least three biologically independent experiments. *P < 0.05, **P < 0.01, ***P < 0.001.

To investigate the function of ADH5 in NSCLC, we performed overexpression of ADH5 in two different NSCLC cell lines (H358 and PC9), validating the expression levels via western blot and qRT**-**PCR (Figure 2a). Overexpression of ADH5 significantly suppressed the proliferation and colony-formation ability of both H358 and PC9 cells (Figure 2b–d). Interestingly, transwell assay showed that overexpression of ADH5 increased cell migration and invasion in both H358 and PC9 cells (Figure 2e and f). Hence, ADH5 overexpression inhibited proliferation but promoted metastasis in NSCLC. The presence of this phenotype is a typical characteristic of cancer cell plasticity [22,23], indicating that ADH5 plays a role in NSCLC cell plasticity. Subsequently, PC9 cells transfected with either the ADH5 overexpression vector or an empty vector were then subcutaneously injected into male BALB/c nude mice. The group with ADH5 overexpression exhibited a notable decrease in tumor volumes and weights compared to the control group (Figure 2g and h).

### 3.3 ADH5 knockdown impaired migration and invasion but enhanced proliferation of NSCLC cells

**Figure 3:**
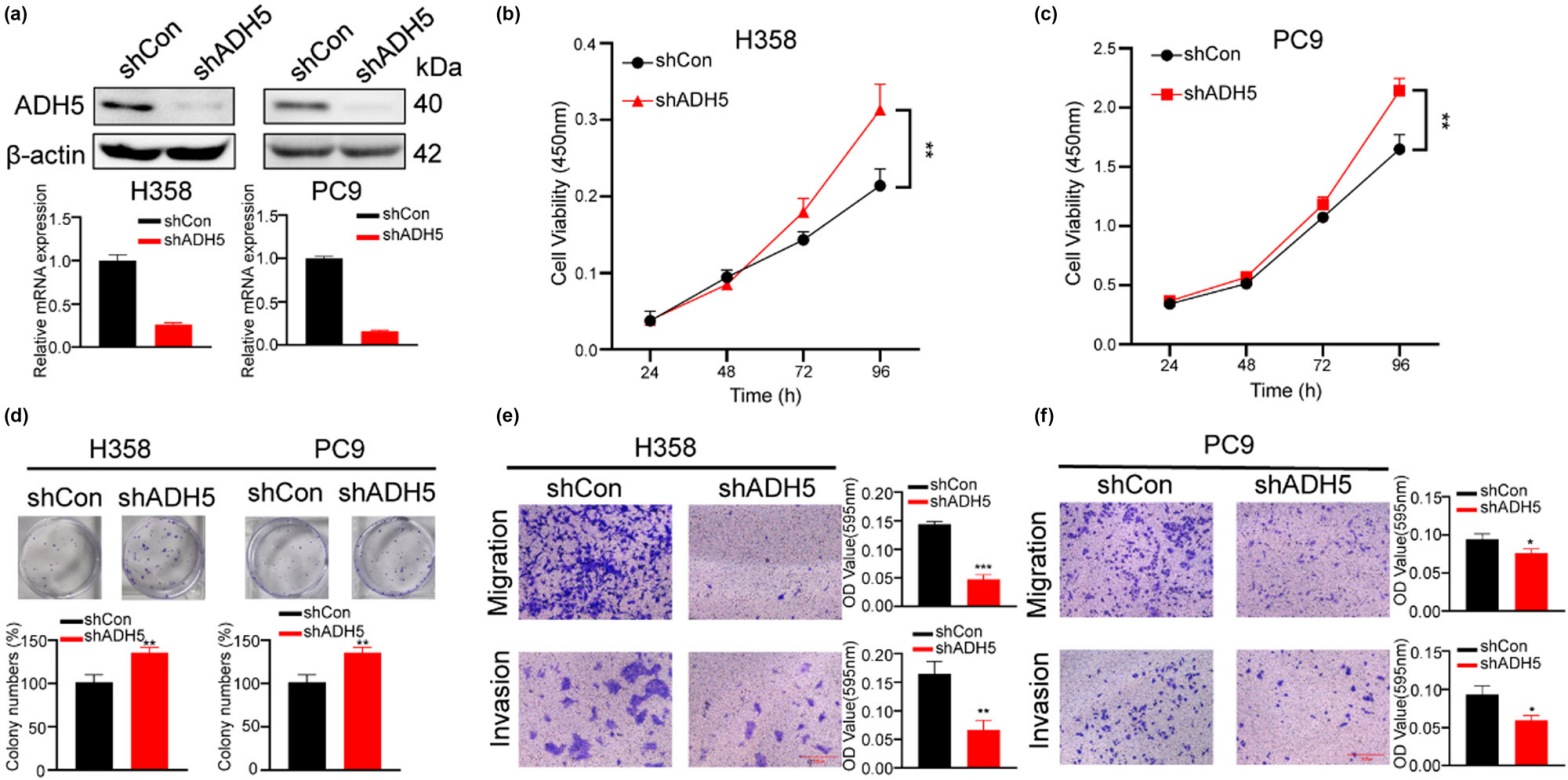
ADH5 knockdown impaired migration but enhanced proliferation of NSCLC cells. (a) Western blot and qRT-PCR showed that ADH5 was efficiently knocked down in H358 and PC9 cells. (b) and (c) CCK8 assay was conducted to measure the proliferation of H358 and PC9 shADH5 cells. The absorbance value was measured at 450 nm. (d) Colony formation was conducted in H358 and PC9 cells lacking ADH5. (e) and (f) Transwell assays were performed to evaluate migration and invasion of H358 and PC9 shADH5 cells. For migration H358 was incubated for 48 h, PC9 was incubated for 24 h. For invasion H358 was incubated for 48 h, PC9 was incubated for 36 h. Data from cells were generated from at least three biologically independent experiments. *P < 0.05, **P < 0.01, ***P < 0.001.

To further assess the impact of reduced ADH5 expression in NSCLC cells, shRNAs targeting ADH5 mRNA were employed to silence the expression of ADH5 in H358 and PC9 cells, which were confirmed with western blot and qPCR (Figure 3a). Consistently, downregulation of ADH5 increased the proliferation and colony-forming capacity but hindered the migration of H358 and PC9 cells (Figure 3b–f).

The advancement of cancer is determined by two key factors: proliferation and metastasis. However, ADH5 plays a “negative” role in cancer proliferation but a “positive” impact on cancer cell migration and invasion which were called metastasis. The discordance in the effects of ADH5 suggests that NSCLC cells are undergoing selective differentiation, emphasizing enhancing metastatic potential while potentially compromising proliferation. This study uncovered the critical involvement of ADH5 in the plasticity of NSCLC cells.

### 3.4 ADH5 activated Smad2/Smad3

**Figure 4:**
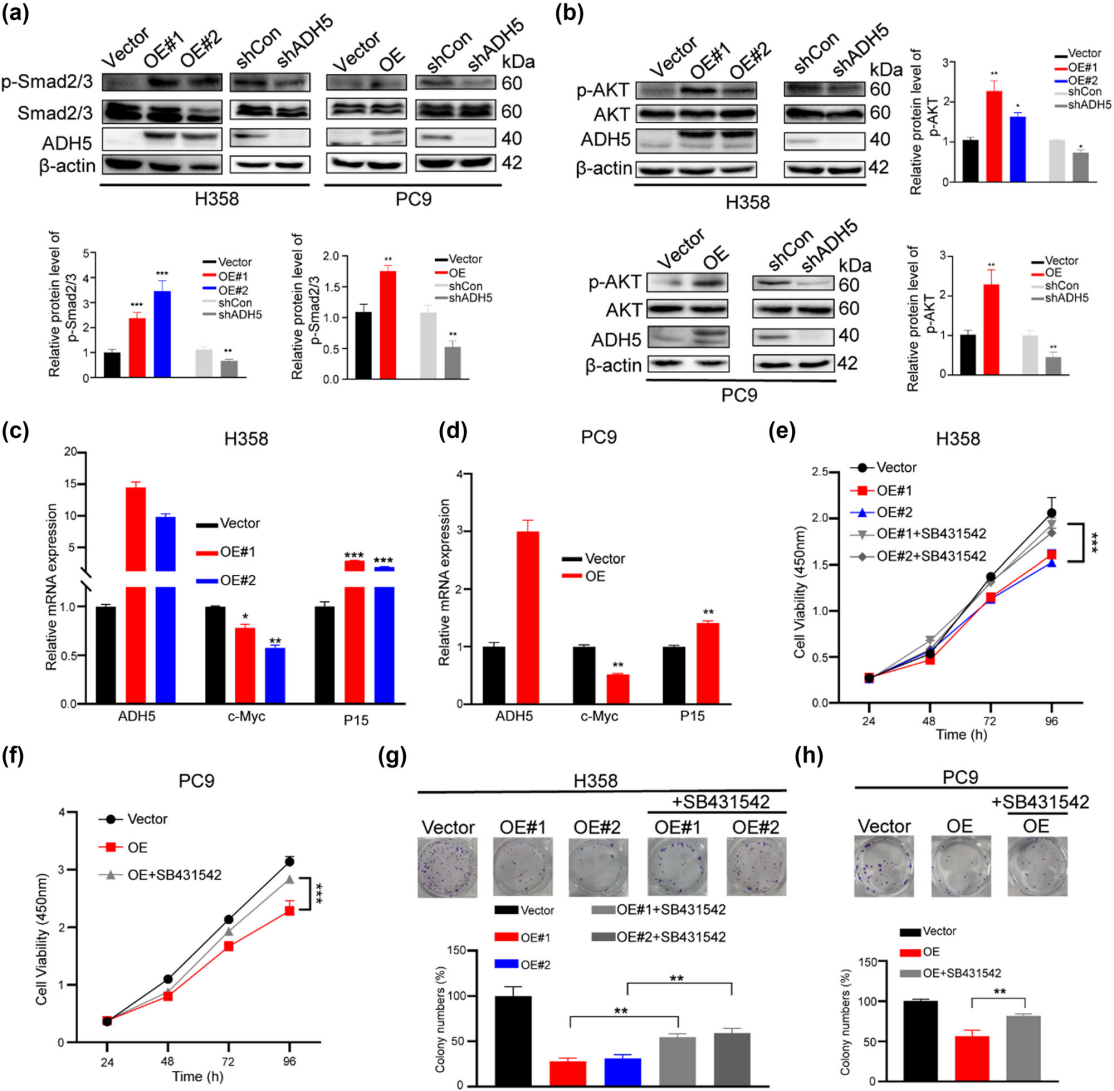
ADH5 activated Smad2/Smad3. (a) Western blot to assess the phosphorylation of Smad2/Smad3 in ADH5 stable transfected H358 and PC9 cells. (b) Western blot to assess the protein expression of ADH5, AKT, and p-AKT in ADH5 transfected stable cell lines. (c) and (d) qRT-PCR was conducted to measure the mRNA levels of c-Myc and P15 in ADH5 stable transfected H358 and PC9 cells. (e) and (f) SB431542 (10 μM) was applied to ADH5 overexpressed cells and cell proliferation was detected by CCK8. (g) and (h) Colony formation was conducted in ADH5 overexpressed cells treated with/without SB431542 (5 μM). Data from cells were generated from at least three biologically independent experiments. *P < 0.05, **P < 0.01, ***P < 0.001.

TGF-β plays a crucial role in regulating cancer cell plasticity, stem cell activity, and tumor progression [24–26]. To clarify whether ADH5 modulates the plasticity of NSCLC cell through the regulation of the Smad2/3 pathway, we detected the phosphorylation levels of Smad2/Smad3 in ADH5-overexpressing stable transfected cells. Our results demonstrated that Smad2/Smad3 activation occurred in cells overexpressing ADH5, while it was inhibited in ADH5 knockdown cells (Figure 4a). AKT phosphorylation, downstream of TGF-β, has been proven to induce cancer cell transdifferentiation, as a regulator of cancer cell plasticity [27,28]. Western blot assays showed that ADH5 overexpression activated AKT while ADH5 knockdown inhibited AKT phosphorylation (Figure 4b). Moreover, ADH5 overexpression suppressed the expression of c-Myc, an important factor for cell proliferation, and upregulated P15, a repressor for cell cycle (Figure 4c and d).

To further validate the modulation of Smad2/3 by ADH5 in NSCLC, TGFbetaR1/ALK5 inhibitor SB431542 was applied. When treated with SB431542, the suppressed proliferation by ADH5 was rescued in H358 and PC9 cells (Figure 4e and f), as well as the colony-formation ability (Figure 4g and h). Taken together, the low expression of ADH5 in NSCLC contributes to the progression of cancer cells by impeding the phosphorylation Smad2/3.

### 3.5 ADH5 upregulated SOX9 and induced EMT

**Figure 5:**
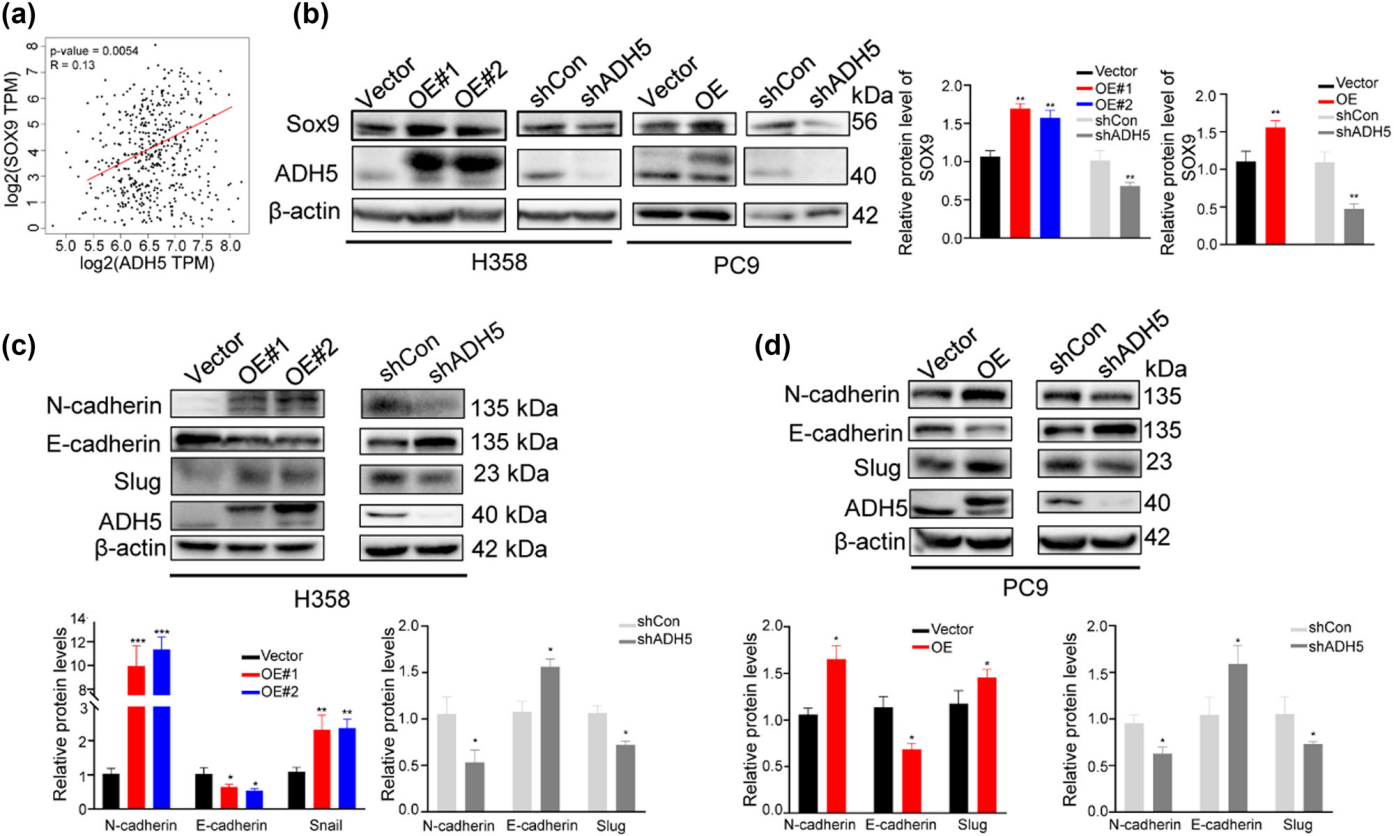
Correlation between ADH5 and SOX9. (a) Scatter plots of correlation between SOX9 and ADH5 expression in lung adenocarcinoma from GEPIA database. (b) Protein level of SOX9 detected by western blot in ADH5 transfected stable cell lines. (c) and (d) Western blot to assess the protein expression of N-cadherin, E-cadherin, Snail, Slug, and ADH5 in ADH5 transfected stable cell lines. Data from cells were generated from at least three biologically independent experiments. *P < 0.05, **P < 0.01, ***P < 0.001.

SOX9 regulates lung cancer cell plasticity, promoting invasion and inhibiting proliferation [29]. Given the analogous role of ADH5 to SOX9 in NSCLC, we explored the crosstalk between ADH5 and SOX9. Analysis showed a positive correlation between ADH5 and SOX9 in NSCLC using the GEPIA database (Figure 5a). Moreover, the overexpression of ADH5 led to an elevation in the SOX9 protein expression, whereas knockdown of ADH5 resulted in a reduction in SOX9 expression (Figure 5b). We then discovered that EMT markers like N-cadherin and Snail showed increased levels with ADH5 overexpression and decreased levels with ADH5 knockdown (Figure 5c and d), which is consistent with the observed effects on cell migration and invasion (Figures 2e, f and 3e, f).

The findings indicated that ADH5 was involved in activating Smad2/Smad3 and SOX9, thereby ultimately promoting EMT. Furthermore, ADH5 contributed to the suppression of c-Myc, leading to the inhibition of cell proliferation. Consequently, ADH5 regulates the plasticity of NSCLC cells by impeding cancer cell growth while facilitating cancer cell metastasis.

## 4 Discussion

**Figure 6:**
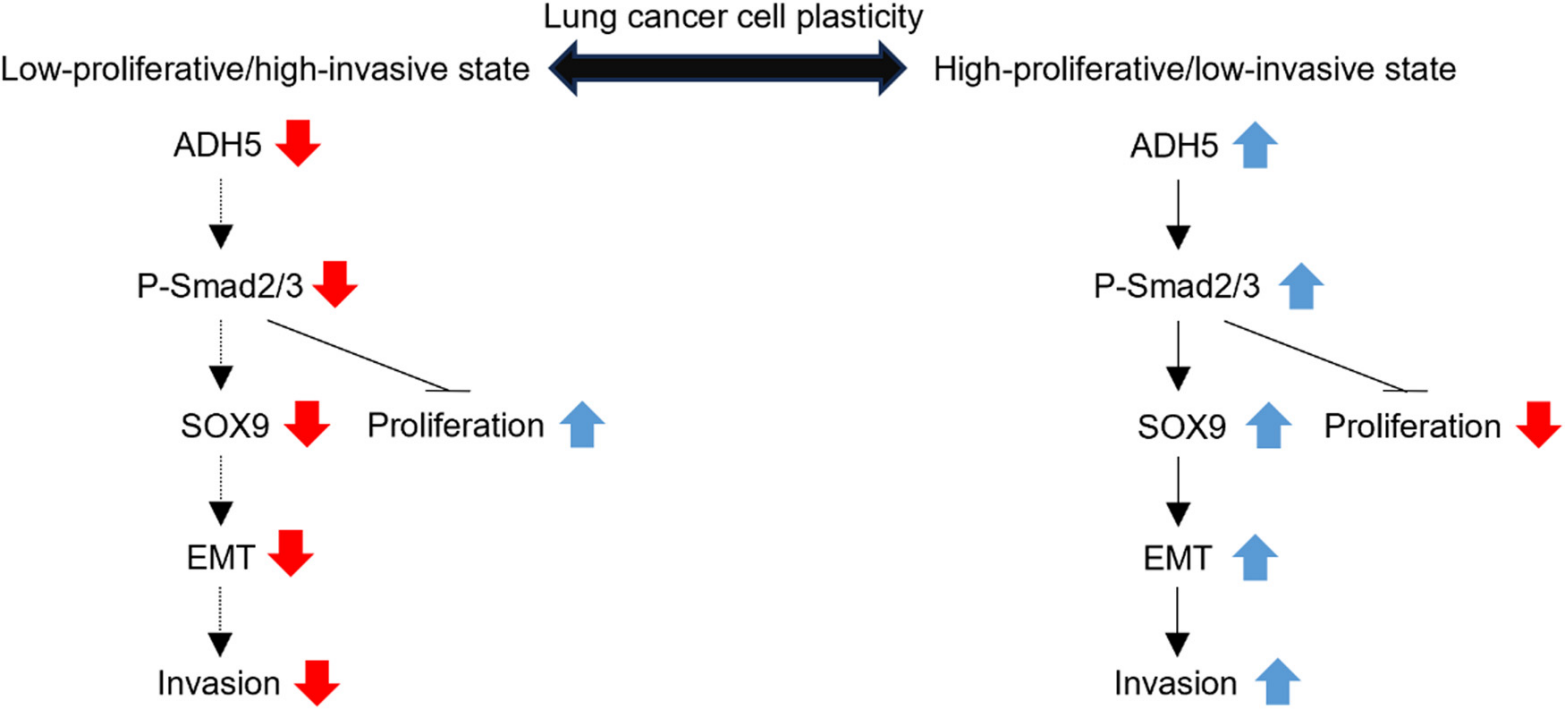
A schematic illustration depicting the regulation of lung cancer cell plasticity by ADH5, where the expression of ADH5 influences the state of lung cancer cells; downregulation of ADH5 suppresses migration but boosts proliferation via Smad2/3, and conversely.

ADH5 has been linked to various histological and pathological processes. As a regulator of protein S-nitrosylation, ADH5 regulates the bioactivity of nitric oxide [2], which is known for its dual roles in tumorigenesis [30]. Depending on its concentration and spatiotemporal profile, nitric oxide exerts diverse effects on several mechanisms within the tumor microenvironment, such as metabolism, cell cycle, DNA repair, angiogenesis, and apoptosis/necrosis [31]. Mice deficient in ADH5 exhibited increased susceptibility to both spontaneous and carcinogen-induced hepatocellular carcinoma due to the S-nitrosylation of DNA repair enzyme O6-alkylguanine-DNA alkyltransferase [10]. ADH5 deficiency induced S-nitrosylation of focal adhesion kinase 1 and sustained tumorigenicity by providing cancer cells with the ability to evade anoikis [8]. It is evident that ADH5 protects against tumor progression. However, S-nitrosylation involves abundant proteins and the overall effect of ADH5 on tumor requires further investigation. In this study, it was observed that ADH5 has a dual role in NSCLC and mainly exerts its function through the Smad2/Smad3 pathway. Overexpression of ADH5 in H358 and PC9 cells hindered proliferation while prompting migration and invasion, which is a typical characteristic of the TGF-β signaling pathway [32]. The overexpression of ADH5 led to the activation of Smad2/Smad3 and EMT in the cells. And SB431542 successfully counteracted the consequence of ADH5 overexpression in NSCLC cells. It was also observed that SOX9 expression shows a strong positive correlation with ADH5 expression in NSCLC cells. These results suggest that ADH5 induces low proliferation and high invasiveness in NSCLC cells through the activation of Smad2/Smad3 and the modulation of SOX9, a key regulator of NSCLC cell plasticity (Figure 6).

The dual role of TGF-β has been implicated in the progression of many types of cancer, inhibiting tumorigenic inflammation during the early stages of carcinogenesis while promoting various phases of the metastatic process, from invasion, dissemination, immune evasive dormancy, and organ-specific colonization [33,34]. Our research uncovered that ADH5 activated Smad2/Smad3, inducing EMT while suppressing c-Myc, thereby enhancing the migratory and invasive abilities of cells and inhibiting proliferation of NSCLC cells. SOX9, a critical regulator of lung cancer cell plasticity, is upregulated by TGF-β both in embryonic development and tumor progression [24,25]. The control of lung cancer cell plasticity involves a balance between SOX2 and SOX9. SOX2 triggers the EpCAM expression and facilitates proliferation, whereas SOX9 drives SLUG transcription and boosts migration [29]. Our findings demonstrated the positive correlation between ADH5 and SOX9, suggesting that ADH5 plays a role in regulating the plasticity of NSCLC cell.

In conclusion, this study identifies ADH5 as a key regulator of NSCLC cell plasticity through its activation of Smad2/Smad3 and upregulation of SOX9. ADH5 shows promise as a potential biomarker for monitoring the progression of NSCLC, and targeting ADH5 expression could offer a novel therapeutic strategy for treating cancer.
